# Estimated cumulative X-ray exposure and additional cancer risk during the evaluation and treatment of scoliosis in children and young people requiring surgery

**DOI:** 10.1007/s43390-021-00314-6

**Published:** 2021-03-03

**Authors:** P. R. Loughenbury, S. L. Gentles, E. J. Murphy, J. E. Tomlinson, V. H. Borse, R. A. Dunsmuir, N. W. Gummerson, P. A. Millner, A. S. Rao, E. Rowbotham, A. L. Khan

**Affiliations:** Centre for Neurosciences, Leeds General Infirmary, Leeds Teaching Hospitals NHS Trust, Great George Street, Leeds, LS1 3EX UK

**Keywords:** Scoliosis, Radiation dose, Cancer risk, Freehand pedicle screws, Cumulative imaging

## Abstract

**Introduction:**

Clinicians and patients must weigh the benefits of radiological imaging against the risks of radiation exposure in the diagnosis and treatment of scoliosis. This report aims to estimate the cumulative absorbed and equivalent dose of radiation in patients undergoing surgical treatment for scoliosis, and to present this as an estimated risk of cancer compared to background radiation levels.

**Methods:**

Retrospective review of estimated absorbed dose on the Computerised Radiology Information System (CRIS^®^). Patients undergoing surgical correction of scoliosis (age ≤ 25) from August 2010 to August 2015 investigated. Estimated absorbed dose [milligrays (mGy)] recorded. Pedicle screws inserted using image intensification. Equivalent dose [millisieverts (mSv)] and additional cancer risk calculated from the National Research Council document ‘Health risks from exposure to low levels of ionising radiation’ (2006).

**Results:**

271 patients identified. Mean age 15 (range 2–25). Mean total absorbed dose 2136 mGy [standard deviation (SD) 1700 mGy]. Mean number of plain spine radiographs was 8 (SD 3) with total 1884 mGy exposure (SD 1609 mGy). Additional dose provided by CT (mean 0.17 episodes), plain chest and abdominal radiographs and image intensification. Mean number of image intensification episodes was 1.1 with mean estimated exposure 180 mGy (SD 238 mGy). Image intensification accounted for 8% of the estimated absorbed dose during treatment. Estimated mean effective dose delivered was 20.952 mSv equating to an additional cancer risk of 0.27–0.45%.

**Conclusion:**

Additional cancer risk from cumulative imaging is small and equivalent to approximately 8 years of natural background radiation. Use of image intensification for pedicle screw insertion is a minor contribution (8%) to the total patient dose.

## Introduction

Radiography is the gold standard imaging technique used during the treatment of patients with scoliosis. Plain spine radiographs are used to confirm the diagnosis, monitor progression of the curve and plan for treatment. Scoliosis is typically diagnosed in childhood or adolescence and is monitored as the spine grows. It is difficult to predict whether a curve will progress during growth and what the rate of progression will be. This leads to multiple repeated imaging episodes during the course of treatment regardless of whether the patient chooses to have surgery or non-operative treatment. Radiographs allow an assessment of the curve magnitude, anatomical location, degree of rotation, and presence of vertebral anomalies, and allow the clinician to make an assessment of the patients remaining skeletal growth. This continued assessment is vital in guiding patients through decisions regarding treatment.

During each imaging episode, a patient receives a dose of ionising radiation and it is not clear what risk is associated with this. Most patients are adolescents and this is a sensitive period for radiation carcinogensis in both breast and thyroid tissue. An elevated standardised mortality ratio for breast cancer has been reported in patients undergoing repeated radiographs in the assessment of spinal disorders; however, this has not been demonstrated in other cancers [1]. In adults, spinal deformity is associated with lower health-related quality of life scores and is a risk factor for a number of other diseases that have an impact on overall health [2]. The elevated standardised mortality ratio for breast cancer may be related to the health effects of residual spinal deformity rather than repeated low-dose radiation exposure.

Clinicians are often asked by patients and their families about the effect of repeated doses of radiation and how this translates to overall risk of problems in adult life. We ask families to be involved in decision-making throughout treatment and, therefore, need to find ways to summarise the evidence relating to risk in a manner that is easy to comprehend. It can be helpful to describe risk in comparison to the risk of background radiation from natural sources as this helps place the risks discussed into a wider health context [3]. This report aims to evaluate the estimated cumulative absorbed and equivalent doses of radiation in patients undergoing surgical treatment for scoliosis and to present this in a clear and reasonable manner as an estimated risk of cancer compared to background radiation levels.

## Methods

Retrospective review of consecutive patients undergoing surgical correction of scoliosis aged under 25 over a 5-year period (August 2010–August 2015). All patients undergoing definitive correction of spinal deformity were included. Procedures to place or lengthen growing rods were excluded. Revision procedures for failure of fusion or add-on above or below a previous correction were also excluded. The number of imaging episodes and mode of each episode was documented.

There was no standardised pathway for imaging episodes but all patients received standing X-rays at presentation and then bending films prior to surgery**.** These films (standing coronal and sagittal views plus bending films) formed the standard pre-operative assessment used for each case. Standing post-operative radiographs were performed immediately after surgery and then at approximately 6 weeks, 3 months, 6 months, one year and two years. Posteroanterior (PA) radiographs were used rather than anteroposterior (AP) radiographs to minimise the dose delivered to breast tissues but no specific protection was used to cover breast tissue during image acquisition. At each whole spine imaging episode, both PA and lateral X-rays were performed.

### Surgical intervention

Definitive correction included both primary surgery and conversion of growing rods to definitive surgery. Surgical approach included both posterior only corrections and two-stage corrections (anterior release and posterior instrumented correction and fusion), either on the same day or staged over the course of a week. Image intensification was used for placement of all screws and this includes an intra-operative level check and ‘screening’ of each screw during placement. In this way, a number of images were obtained using radioscopy following the insertion of the screw, rather than a single check after placement. The total dose delivered during the case was considered to be one ‘image intensification episode’. Where surgery included a staged procedure this was counted as two separate imaging episodes regardless of whether the stages were on the same day or not. Intra-operative monitoring (both somatosensory and motor evoked potentials) was used throughout all procedures.

### Absorbed dose

The absorbed dose [milligrays (mGy)] is an estimation of the dose delivered during a single episode and is a measure of potential tissue damage. This was recorded on the Computerised Radiology Information System (CRIS^®^). The dose for each episode during the treatment pathway that involved ionising radiation was collected. This is a much larger dose than the entrance surface dose and so is used to provide a measure of proportion of does in each imaging episode rather than a measure of the dose received by the patient and the associated risk.

### Equivalent dose

The equivalent dose [milliSieverts (mSv)] considers the biological effects of radiation and the radiosensitivity of different types of tissues. The equivalent dose is calculated using the age of the patient and number and mode of imaging episodes. This calculation was performed using an online risk calculator [4]. This includes estimates of average effective dose from each imaging episode [5] and the lifetime attributable risk of cancer from exposure to background radiation suggested by the United States National Research Council [6].

## Results

271 consecutive patients included with a mean age of 15 (range 2–25). 226 were female and 45 were male. There were 244 stand-alone posterior instrumented scoliosis correction procedures and 27 patients underwent a staged anterior release and posterior instrumented scoliosis correction (25 with both stages on the same day and 2 cases where stages were performed one week apart). An image intensifier episode was attached to each stage leading to a mean of 1.1 image intensifier episodes per patient. None of the patients had been treated with a brace prior to surgery and this reflects the treatment philosophy of the unit.

**Fig. 1:**
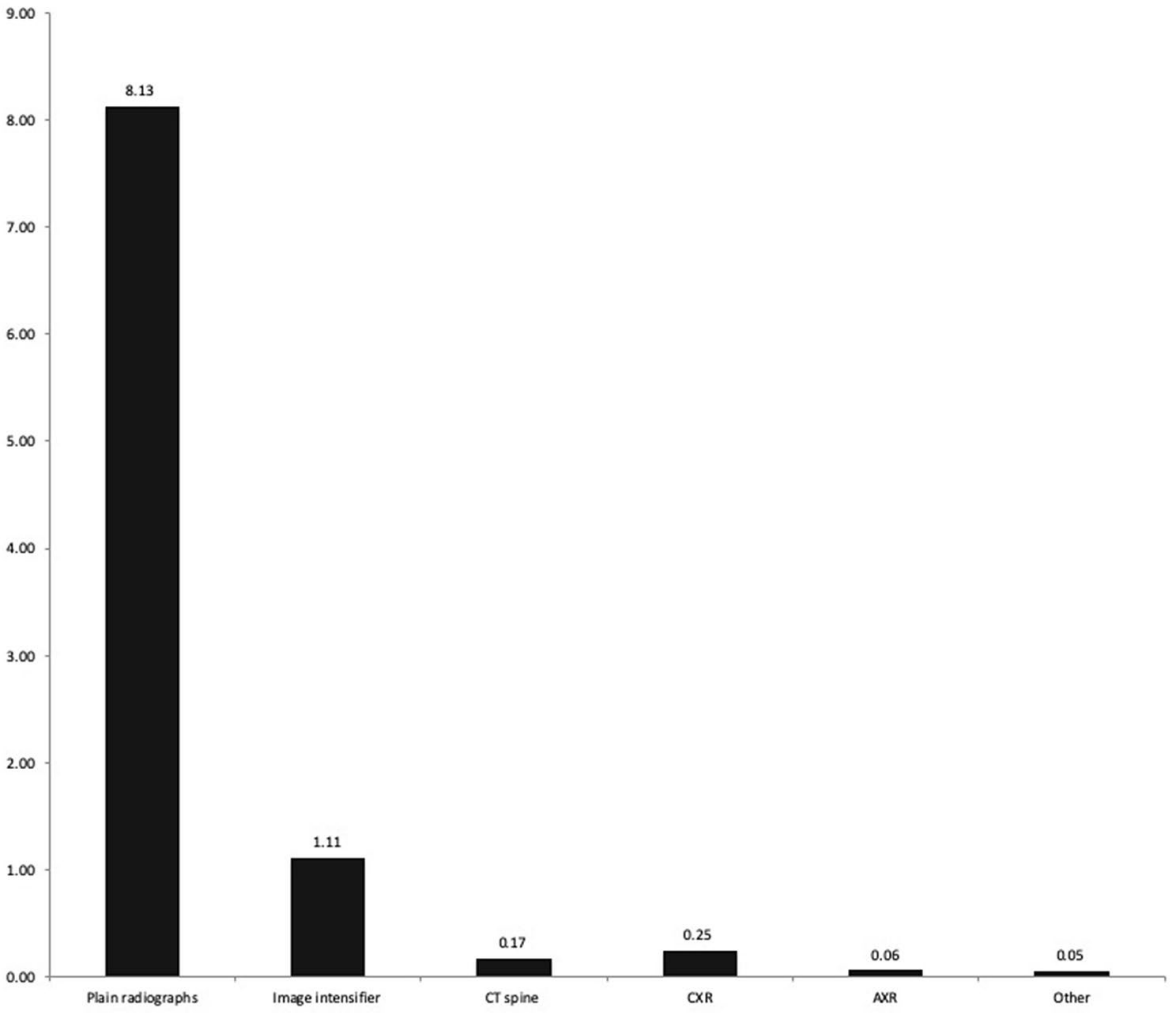
Mean number of imaging episodes during treatment pathway

Figure 1 shows the mean number of imaging episodes for each imaging modality. Standing plain films were most frequently used with a mean 8.13 episodes (SD 3.08). Additional dose was provided by CT (mean 0.17 episodes, SD 0.46), plain chest (CXR) and abdominal (AXR) radiographs.

**Fig. 2:**
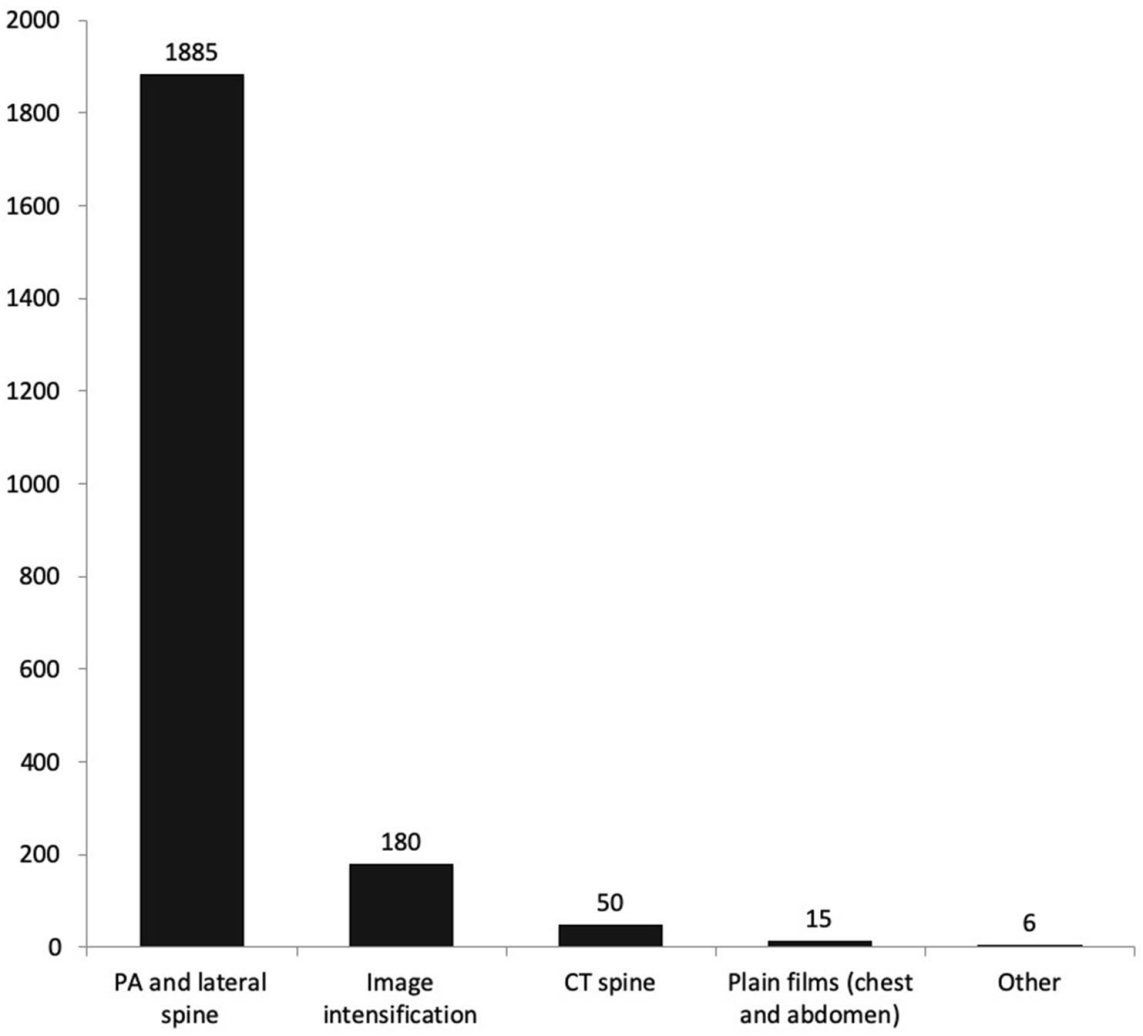
Mean total absorbed dose (mGy) during treatment pathway

Figure 2 shows the estimated mean absorbed dose for each mode of imaging. Cumulative mean absorbed dose was 2136 mGy (SD – 1700 mGy). Mean dose for plain radiographs was 1884 mGy (SD 1609 mGy) and for image intensification was 180 mGy (SD 238 mGy). Image intensification accounted for 8% of the estimated absorbed dose during treatment.

Estimated mean equivalent dose was 20.952 mSv (SD – 14.23 mSv). This equates to an additional cancer risk of 0.27% for male patients and 0.45% for female patients.

## Discussion

Assessment of risk during medical imaging episodes involving ionising radiation is complex and imprecise. It is particularly difficult when treating patients with scoliosis because there is no consistent regime for the number and type of imaging episodes that are required. Standard radiographs at time of presentation include a standing coronal and sagittal whole spine image that includes the shoulders, pelvic brim and the centre of the femoral heads [7]. Once a diagnosis has been made, it is difficult to predict whether a curve will progress and what the rate of progression will be. There are, therefore, multiple repeated imaging episodes during the course of treatment to help guide treatment strategy. This will be the same whether or not the patient chooses to have surgery or non-operative treatment. Historical follow-up studies have suggested that between 20 and 42 imaging episodes occur during treatment [1, 8, 9] and a more recent study reported a mean of 16 episodes [10]. In the current study, the number of episodes was much lower (mean 8.13) and this is probably due to the fact that there was no bracing for any of the patients in our cohort. There may also be a general move towards limiting X-ray exposure where possible in more recent studies due to worries over associated risk. In our study some patients received CT imaging during their inpatient stay. These were not routine imaging episodes but were clinically indicated and we feel including them provides a more relevant estimation of risk for the patient cohort. However, CT scanning is not required for most patients and this may be a confounding factor when interpreting the results. In addition, there may be occasions of additional exposure that fall outside of our standard protocol (such as films from other centres that refer to our hospital) and these have not been captured in the study.

The absorbed dose provides a measure of potential tissue damage. In the present study, this was estimated from the dose delivered by the apparatus and, therefore, takes into account machine specific parameters such as field size, tube voltage, tube current and exposure time. It does not consider the distance between the generator and the tissues or patient parameters such as age, height, weight, or body mass index. It is, therefore, a blunt estimation and should be used to consider the contribution of each imaging modality to the overall dose only—not the total dose absorbed by tissues within the image area. The estimated entrance surface (skin) dose is more relevant and can be calculated using these parameters (using a standardised back scatter factor) or recorded using a calibrated dosimeter placed on the patient’s skin. This still provides an inexact estimation as the absorbed dose will vary across all exposed tissues. A standard series of two standing whole spine images (coronal and sagittal planes) can be considered to involve an absorbed dose of 2.88 mGy [11]. The doses recorded in the current study are significantly higher and varied between 8.5 and 2011 mGy. It is likely that the dose absorbed by the tissues is similar during each imaging episode and the variation seen in the current study was required to enable an adequate image to be generated. The measured dose can only be used to identify the proportion of radiation from each modality—with the majority being attributed to plain films (Figs. 1, 2).

The equivalent dose calculation is independent of the absorbed dose and based on the number and type of imaging episodes and the age of the patient [5, 6]. This provides an estimation of the biological effect of the radiation. The mean dose reported across all imaging episodes in our study (20.95 mSv) is equivalent to 8 years of natural background radiation dose. This is in keeping with that reported by Simony et al. [10] who estimated a cumulative dose of 13.04–22.82 mSv. The dose attributable to the use of an image intensifier was much lower (1.68 mSv) and was equivalent to 6 months of natural background radiation dose. This finding is unique to the present study as it is the first to consider the relative contribution of the use of image intensifier during screw placement on the overall dose of ionising radiation. It is certainly useful to the surgeon to know that using intra-operative imaging is only a minor contribution to the overall dose.

Low-dose digital X-ray devices are increasingly being used to diagnose and monitor skeletal deformity. These have not been considered in the current study. The EOS^®^ 2D/3D X-ray imaging system uses slot scanning technology based on multiwire proportion chambers, for which Georges Charpak won the Nobel Prize in Physics in 1992 [11]. This technology leads to an entrance surface dose that is five to six times lower than plain radiographs [12]. Whilst this technology is gaining in popularity, it is not readily available to every unit. Use of low-dose systems would be likely to reduce both the estimation of dose and the estimation of additional cancer risk. Intra-operative navigation has also not been considered in the current study. Use of a navigation system would certainly have an impact on intra-operative radiation exposure. However, with numerous emerging technologies to aid intra-operative navigation, it seems preferable to consider overall risk without navigation technology and to allow clinicians to factor in the additional risk associated with the system they choose to use.

### Estimation of additional cancer risk

Theoretical estimations of cancer risk are useful to help both clinicians and patients consider the effect of radiation dose. There are two commonly employed methods for estimating risk—measurements of cancer relative risk ratios in cohorts of patients with scoliosis or estimations based on data regarding radiation exposure (linear no threshold estimations) [13].

Assessing the relative risk ratio is reasonably robust method but relies on a large data set and a number of assumptions. There are two major scoliosis cohorts reported in the literature. Hoffman et al. [8] initially reported on a pilot cohort of 1030 women treated in Minneapolis between 1935 and 1965 who had an increased standardised incidence ratio for breast cancer of 1.82 [confidence interval (CI) 1.0–3.0]. This was expanded to include 5573 women diagnosed with scoliosis at 14 centres in the United States of America (US Scoliosis Cohort Study) between 1912 and 1965 [1, 9]. In this cohort, cancer mortality was 8% higher than expected and the standardised mortality ratio for breast cancer was significantly elevated at 1.68 (CI 1.38–2.02). However, an increased standardised mortality ratio was not demonstrated in other cancers. The second cohort was reported by Simony et al. [10] and includes 215 scoliosis patients in Denmark treated between 1983 and 1990. In this group, there was also an increased standardised incidence ratio for breast or endometrial cancer of 4.8 (CI 2.3–5.8). An increased risk is demonstrated in each of these cohorts but it is not clear whether this is due to repeated radiographs or another factor relating to the diagnosis, investigation or treatment of scoliosis. In adult spinal deformity, the presence of a scoliosis is associated with lower health-related quality of life scores and an overall increased mortality due to other conditions such as atherosclerosis [2]. It may be that elevated cancer risk in these cohorts represents the increased health burden of spinal deformity rather than a side effect of repeated low-dose radiation exposure.

The present study uses a linear no threshold estimation. This system recognises the lack of data for low-dose exposure and so attempts to provide an estimate of cumulative dose and use this to estimate risk. The technique was employed by Levy et al. [14], who estimated the additional cancer risk in 2039 patients undergoing treatment for scoliosis between 1965 and 1979 in Montreal, Canada. They reported 42–238 excess cancer cases per 100,000 for women and 14–79 excess cases per 100,000 for men.

When an estimated risk is presented as a ratio or an increase in the number of cases per 100,000 it can be difficult to grasp. We know that using a comparison approach is much more accessible for patients and their families [3] and so this is how we have chosen to report risk in our series. We know that the risk associated with one whole spine standing X-ray (PA and lateral) is approximately the same as a transoceanic return flight [3]. The risk of cumulative has not been reported previously but, however, risk is reported, the overall risk of cumulative imaging is low and is equivalent to an additional lifetime cancer risk of 0.27% for men and 0.45% for women.

## Conclusion

Additional cancer risk from cumulative imaging during surgical treatment for scoliosis is small and equivalent to approximately 8 years of natural background radiation. This includes initial assessment, surgical treatment and follow-up imaging until at least 2 years after surgery. Use of image intensification for pedicle screw insertion is a minor contribution (8%) to the total patient dose and is equivalent to approximately 6 months of natural background radiation.

## Data Availability

Not applicable.

## Availability of data and material

Available at request.
